# Prognostic value of venous thromboembolism in patients with advanced pancreatic cancer: a systematic review and meta-analysis

**DOI:** 10.3389/fonc.2024.1331706

**Published:** 2024-02-08

**Authors:** Kaifeng Su, Ruifeng Duan, Yang Wu

**Affiliations:** ^1^ Medical Faculty of Ludwig‐Maximilians‐University of Munich, University Hospital of LMU Munich, Munich, Germany; ^2^ Department of Gastroenterology and Digestive Endoscopy Center, The Second Hospital of Jilin University, Changchun, China; ^3^ Pancreas Center, The First Affiliated Hospital of Nanjing Medical University, Nanjing, China

**Keywords:** venous thromboembolism, advanced pancreatic cancer, meta-analysis, prognosis, VTE

## Abstract

**Objective:**

This study aimed to investigate the relationship between the incidence of VTE and the prognosis of patients with advanced pancreatic cancer, as there is currently a lack of systematic research on this topic, despite the prevalence of venous thromboembolism (VTE) in patients with pancreatic cancer.

**Methods:**

Databases including PubMed, Embase, Web of Science, and Cochrane Library were searched until April 9, 2023, to identify studies that explored the relationship between VTE and the prognosis of advanced pancreatic cancer. Duplicate publications, studies without full text or sufficient information for data extraction, animal experiments, reviews, and systematic reviews were excluded. The extracted data were analyzed using STATA 15.1.

**Results:**

The pooled results indicated a significant association between the incidence of VTE and poorer overall survival (HR=1.38, 95% CI: 1.24 - 1.53, p < 0.001) and disease-free survival (HR=2.42, 95% CI: 1.94 - 3.04, p < 0.001) among patients with advanced pancreatic cancer. Additionally, early VTE showed a significant impact on overall survival (HR=2.03, 95% CI: 1.33 - 3.12, p = 0.001), whereas late VTE did not demonstrate a significant association with poor overall survival (HR=1.22, 95% CI: 0.96 - 1.54, p = 0.099).

**Conclusions:**

This study found that advanced pancreatic cancer patients with VTE had poorer overall and disease-free survival than those without. Meanwhile, the patients with early VTE had a significantly poorer prognosis, whereas late VTE did not. The findings highlight the importance of timely detection of VTE for patients with advanced pancreatic cancer patients and offer a partial theoretical basis for future clinical endeavors.

**Systematic review registration:**

https://www.crd.york.ac.uk/prospero/display_record.php?ID=CRD42023427043, identifier CRD42023427043.

## Introduction

Pancreatic cancer is a deadly disease with dismal prognoses and a rising incidence (1). It has surpassed breast cancer to become the third leading cause of cancer-related death in the United States. Worryingly, it is expected to surpass colorectal cancer and become the primary cause of cancer-related mortality by, 2040, trailing only lung cancer (2). Unfortunately, patients with pancreatic cancer still face bleak prognoses, with an overall survival rate of only 5% across all stages of this disease. Those with localized disease have a slightly higher survival rate of 20%, while patients with distant metastasis experience a survival rate of just 1% to 2% (3). Therefore, identifying prognostic risk factors in advance can enable physicians to implement timely treatment and preventive measures more effectively.

Venous thromboembolism (VTE) is a common complication among cancer patients, It occurs at a four to six-fold higher rate among cancer patients compared to those without cancer, which causes increased morbidity, mortality, and healthcare costs (4, 5). Cancer type and systemic chemotherapy are key risk factors for VTE development in cancer patients and pancreatic cancer is strongly associated with thrombotic problems (6). Multiple studies have demonstrated that advanced tumor stages pose a greater risk for VTE compared to early tumor stages (7–9). In pancreatic cancer patients receiving chemotherapy for advanced disease, the incidence of VTE can reach 40% (10). Notably, Blom et al. (11) recently reported a high incidence of VTE in patients with locally advanced or metastatic pancreatic cancer patients, with a 60-fold increased risk of venous thrombosis compared to the general population and a cumulative risk of nearly 10%. Previous epidemiological investigations have estimated the incidence of VTE in patients with metastatic pancreatic cancer to be as high as 41% (12). As the majority of patients with pancreatic cancer are diagnosed with advanced or metastatic disease, rendering surgery as an impractical curative option (13). Therefore, exploring the relationship between VTE and the prognosis in patients with advanced pancreatic cancer is of paramount importance. However, However, no consensus has been reached on whether VTE influences the prognosis of advanced pancreatic cancer. Some studies have indicated that the incidence of VTE is associated with poor prognosis in patients with pancreatic cancer (14–19), while others have reported no apparent impact caused by VTE on overall survival among patients with advanced pancreatic cancer (20–23).

Given these divergent results, we conducted a systematic literature review and meta-analysis to evaluate the relationship between VTE incidence and survival in patients with advanced pancreatic cancer. We aimed to provide a theoretical foundation for ascertaining and preventing VTE in these patients during their hospitalization.

## Methods and materials

This study protocol is registered on PROSPERO (CRD42023427043). We followed the Systematic Reviews and Meta-analyses guidelines.

### Literature search and inclusion criteria

We screened studies in PubMed, Embase, Web of Science, and Cochrane Library up to April 9, 2023. The following terms were used for the literature search: (“Pancreatic Neoplasms” [Mesh] OR “Pancreatic Cancer” [Mesh] OR “Pancreatic adenocarcinoma” [Mesh] OR “Pancreatic ductal adenocarcinoma” [Mesh] et al.) AND (“Venous Thrombosis” [Mesh] OR “Venous Thromboembolism” [Mesh] OR “Deep-Vein Thrombosis” [Mesh] et al.). Details of the literature search are presented in **Supplementary Table 1**.

Inclusion criteria are as follows: I) The studies included in the analysis consisted of both retrospective and prospective cohort studies and case-control studies. II) Participants enrolled in the studies were diagnosed with advanced pancreatic cancer, which included stage III/IV, locally advanced, metastasis, and unresectable cases. III) The exposure cohort consisted of individuals who experienced any type of VTE after the diagnosis of advanced pancreatic cancer, while the compare cohort included participants without VTE after the diagnosis of advanced pancreatic cancer (early VTE was classified as the occurrence before therapy, and late VTE was classified as the occurrence of it after treatment). IV) The primary outcome was overall and disease-free survival among participants.

Exclusion criteria are as follows: I) animal experiments, II) meta-analyses, reviews, conference abstracts and letters, III) randomized controlled studies (it aimed to assess the efficacy of the intervention, hence the lack of data on the prognosis of VTE in pancreatic cancer), IV) cross-sectional studies (its purpose was to evaluate epidemiologic investigations of a specific period. There is no match with our study which requires long term follow up).

### Data extraction

Two researchers independently performed the literature search, filtering, and information extraction. In case of uncertainties or disagreements, a third party was consulted before the final decision. The data extraction involved gathering information on authors, publication year, study region, study type, sample size, age, gender, tumor stage, therapy, and outcome measures such as overall survival (OS) and disease-free survival (DFS). For studies providing data on OS and DFS, these two outcomes were expressed as hazard ratio (HR) with 95% confidence interval (CI). For articles presenting survival curves without explicit hazard ratios (HR) and 95% confidence intervals (CI), the Engauge Digitizer software (24) was utilized to extract the HR with 95% CI.

### Literature quality assessment

Two researchers independently evaluated the quality of included studies using the Newcastle-Ottawa Scale (NOS) for cohort studies (25). Cohort studies were assessed using the NOS from three perspectives: the selection of study groups, the comparability of the groups, and the ascertainment of either the outcome of interest. A study was awarded up to nine scores, where a score of no less than seven indicated high quality and a score of less than seven suggested low quality. Any disagreements over the decision on study quality were resolved by discussion with a third reviewer until a consensus was reached.

### Statistical analysis

The data extracted from included studies were analyzed using the software STATA version 15.1 (Stata Corporation, College Station, TX, USA). OS and DFS were expressed as HR (95% CI). Heterogeneity across studies was assessed using Cochrane’s Q test and the *I* ^2^ statistic. P ≥ 0.1 and *I* ^2^ ≤ 50% indicated no significant heterogeneity, and then a fixed-effects model would be utilized for data analysis; otherwise, a random-effects model would be employed. Additionally, sensitivity analysis was performed to test the stability of the results, and the publication bias was investigated using Begg’s and Egger’s tests. A p-value < 0.05 suggested statistical significance.

## Results

### Literature search

**Figure 1:**
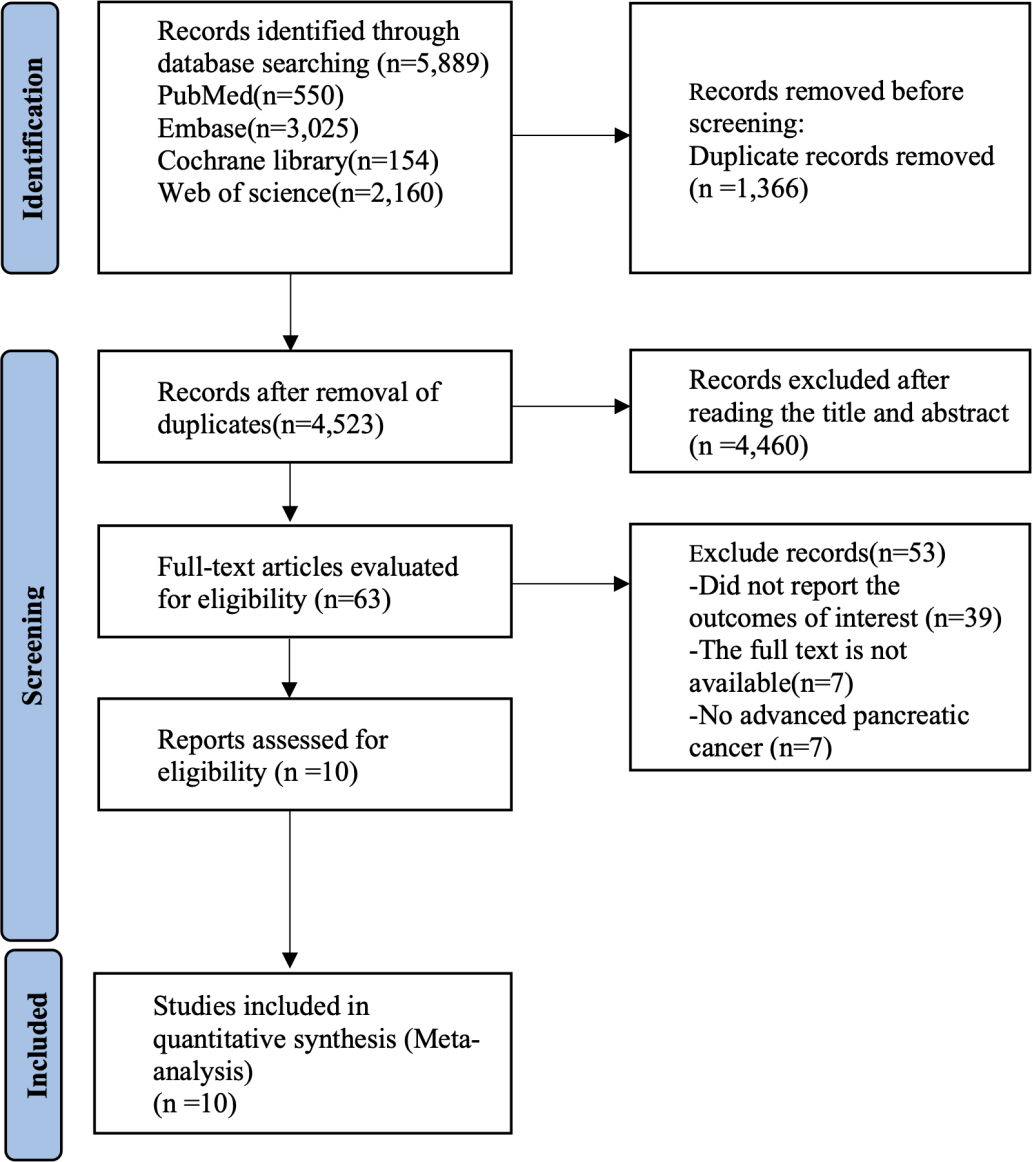
Flow diagram for selection of studies.

The initial literature search of databases produced 5,889 studies in total. The screening of titles and abstracts excluded 1,366 duplicates and another 4,460 articles. The review of the remaining 63 studies resulted in the removal of 53 studies failing to satisfy the eligibility criteria. Finally, ten studies were determined to be eligible for meta-analysis. The literature screening process is summarized in **Figure 1**.

### Baseline characteristics and quality of the included studies

This meta-analysis targeted ten cohort studies comprising 3,145 patients in total (14–23). The sample sizes in these studies ranged from 170 to 838. Among the ten studies, 1,903 Asian patients (17, 18, 20, 22, 23) and 1,242 European and American patients (14–16, 19, 21) were enrolled. The majority of the patients were middle-aged and elderly individuals diagnosed with advanced pancreatic cancer. Treatment modalities included chemotherapy (14–16, 18–20, 22, 23) or chemoradiotherapy (17, 21). Our research aimed to assess the predictive value of VTE in patients with advanced pancreatic cancer by combining OS and DFS data. Six studies provided the HR with 95% CI directly (14–18, 22), and the rest four only presented survival curves that required independent extraction (19–21, 23). NOS scores for studies were above seven, indicating their quality satisfying the eligibility criteria. Study characteristics are summarized in **Table 1**.

**Table 1:** Baseline Characteristics and Quality Assessment of the Included Studies.

Author	Year	Start Duration	Country	Sample Size	Gender (male/female)	Age	Tumor Stage	Therapy	Time of VTE	Extraction method	Outcome	NOS Score
Mandala et al. (14)	2007	2001.12 – 2004.12	Italy	227	121/160	63 (38 -82)	locally advanced / metastatic	Chemotherapy	/	HR (95% CI)	OS/PFS	8
Lambert et al. (15)	2016	2001.01 – 2011.05	France	142	87/55	61 (28 -89)	locally advanced / metastatic	Chemotherapy	/	HR (95% CI)	OS	9
Kruger et al. (21)	2017	2002 -	Germany	299	102/70	63 (40 - 83)	locally advanced / metastatic	Chemo and radiotherapy	Before and after therapy	Survival curve	OS	8
Chen et al. (22)	2018	2010 - 2016	Chian	838	497/341	62 (23 -89)	Stage III/IV	Chemotherapy	Within and beyond 1.5 months after therapy	HR (95% CI)	OS	8
Kim et al. (23)	2018	2005.01 – 2015.12	Korea	216	131/85	63 (38 -83)	metastatic	Chemotherapy	Within and beyond 30 days after therapy	Survival curve	OS	8
Yoon et al. (20)	2018	2006.01 – 2012.12	Korea	505	294/211	65 (32 -88)	advanced	Chemotherapy	/	Survival curve	OS	8
Barrau et al. (16)	2021	2010 - 2019	France	174	96/78	67 (60 -75)	locally advanced / metastatic / recurrence after tumor resection	Chemotherapy	Before therapy	HR (95% CI)	OS/PFS	9
Yamai et al. (17)	2022	2017.04 – 2020.03	Japan	174	90/84	63 (45 -79)	unresectable metastatic	Chemo and radiotherapy	After therapy	HR (95% CI)	OS/PFS	9
Jeong et al. (18)	2023	2011.01 – 2020.12	Korea	170	99/71	64 (56 -72)	locally advanced / metastatic	Chemotherapy	After therapy	HR (95% CI)	OS	9
Laderman et al. (19)	2023	2010 - 2016	USA	400	208/192	66 (27-90)	metastatic	Chemotherapy	/	Survival curve	OS	8

“/”: not provided in the article.

### Overall survival

**Figure 2:**
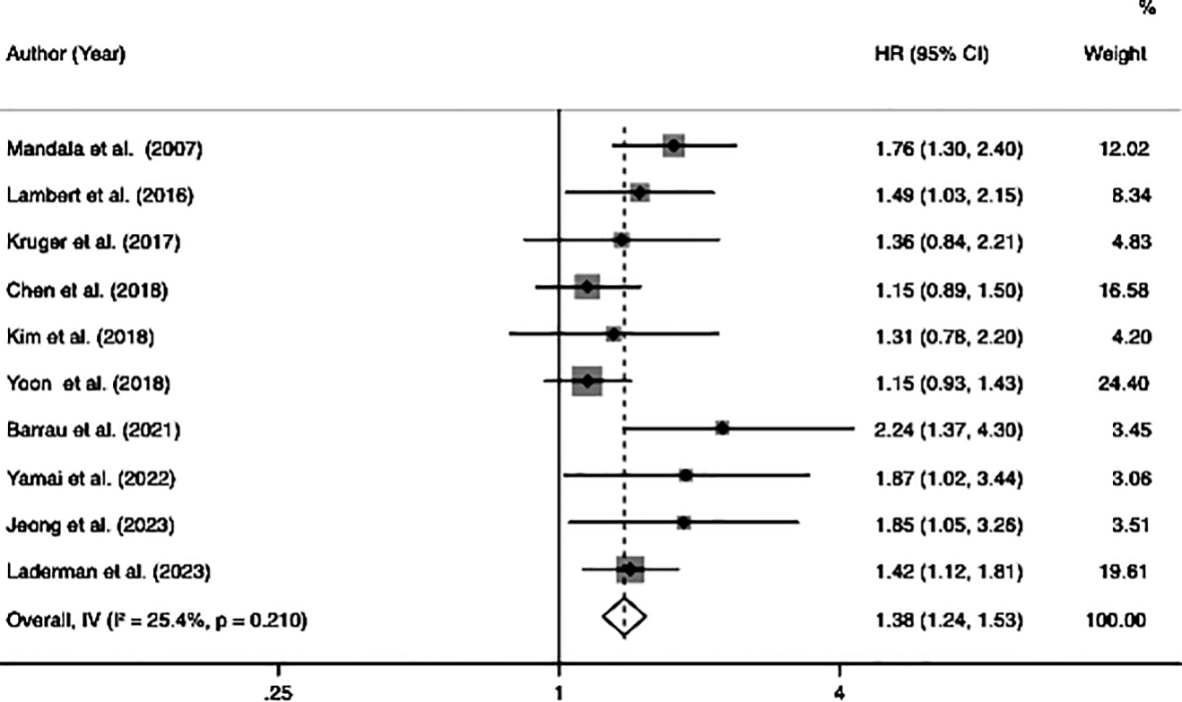
Forest plots of 10 studies examining the association between VTE and the overall survival of patients with advanced pancreatic cancer.

Ten studies investigated the relationship between VTE and OS, involving 3,145 patients (605 patients with VTE and 2,540 patients without VTE). Since there was no significant heterogeneity across the ten studies (*I* ^2^ = 25.4%, p = 0.21), a fixed-effects model was employed to conduct the meta-analysis whose results showed that patients with VTE were significantly associated with poor OS (HR=1.38, 95% CI: 1.24 - 1.53, p < 0.001) (**Table 2**; **Figure 2**).

**Table 2:** The details of results after analysis.

Subgroups	Independent cohorts	Sample size	I^2^	P	HR (95%CI)	P value	Egger	Begg	After Trim and Fill analysis (P)
Overall survival	10	3145	25.40%	0.21	1.38 (1.24 - 1.53)	< 0.001	0.041	0.107	< 0.001
Early VTE	4	1278	75.80%	0.006	2.03 (1.33 - 3.12)	0.001			
Late VTE	5	1531	39.80%	0.156	1.22 (0.96 - 1.54)	0.099			
Extraction method									
HR (95% CI)	6	1725	37%	0.16	1.51 (1.29 - 1.76)	< 0.001			
Survival curve	4	1420	0%	0.629	1.28 (1.10 - 1.48)	0.001			
Region									
Europe and America	5	1242	0%	0.543	1.55 (1.33 - 1.81)	< 0.001			
Asia	5	1903	11%	0.343	1.24 (1.07 - 1.43)	0.005			
Therapy									
Chemotherapy	8	2672	36.70%	0.136	1.37 (1.22 - 1.53)	< 0.001			
Chemo and radiotherapy	2	473	0%	0.422	1.54 (1.05 - 2.25)	0.026			
Disease-free survival	3	575	2.10%	0.36	2.42 (1.94 - 3.04)	< 0.001			

### Subgroup analysis

Due to the heterogeneity of the research results, we performed a subgroup analysis based on the timing of the onset of VTE, extraction method, different regions, and types of therapy.

The OS among participants with early or late VTE was compared with that among those without VTE. The analysis results demonstrated that patients with early VTE were significantly associated with poor OS (HR=2.03, 95% CI: 1.33–3.12, p=0.001) (**Table 2**; **Supplementary Figure S1**). However, patients with late VTE were not significantly associated with poor OS (HR=1.22, 95% CI: 0.96–1.54, p=0.099) (**Table 2**; **Supplementary Figure S2**). The analysis of the HR (95% CI) that studies directly reported showed that patients with VTE were significantly associated with poor OS (HR=1.51, 95% CI: 1.29–1.76, p<0.001). Moreover, the same trend was noted in the analysis of the HR (95% CI) extracted by the software based on survival curves in studies of interest (HR=1.28, 95% CI: 1.10 - 1.48, p=0.001) (**Table 2**; **Supplementary Figures S3**, **S4**). Furthermore, the subgroup analysis based on the study population demonstrated s significant association between VTE and poor OS in both Europe and America (HR=1.55, 95% CI: 1.33–1.81, p < 0.001), as well as in the Asian region (HR=1.24, 95% CI: 1.07–1.43, p=0.005) (**Table 2**; **Supplementary Figures S5**, **S6**). Additionally, the analysis results revealed that VTE was significantly associated with poor OS in patients who underwent chemotherapy (HR=1.37, 95% CI: 1.22–1.53, p < 0.001) or chemoradiotherapy (HR=1.54, 95% CI: 1.05–2.25, p=0.026) (**Table 2**; **Supplementary Figures S7**, **S8**).

### Disease-free survival

**Figure 3:**
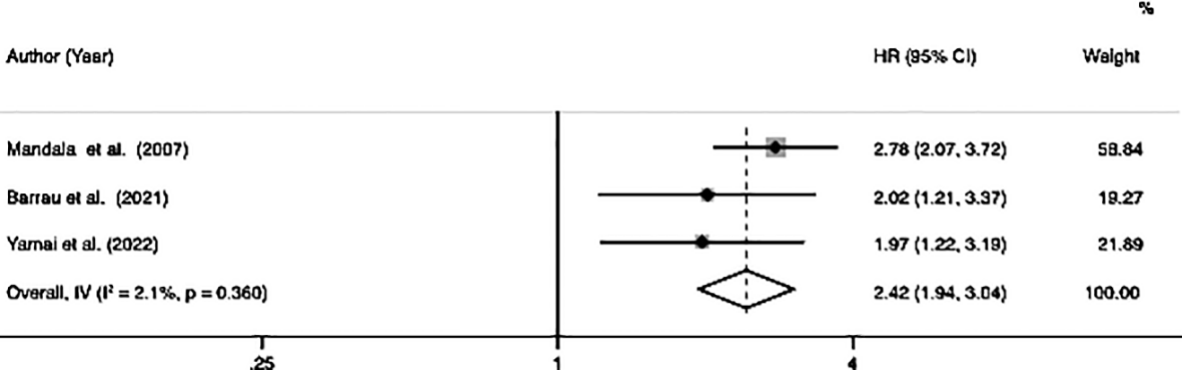
Forest plots of 3 studies examining the association between VTE and disease-free survival of patients with advanced pancreatic cancer.

Among the ten studies, three studies involving 575 patients (122 patients with VTE and 453 patients without VTE) indicated a correlation between VTE and DFS. The meta-analysis of these three studies was performed using a fixed-effects model due to a lower degree of heterogeneous (*I* ^2^ = 2.1%, p=0.36). The analysis results showed that patients with VTE were significantly associated with poor DFS (HR=2.42, 95% CI: 1.94 - 3.04, p < 0.001) (**Table 2**; **Figure 3**).

### Sensitivity analysis

**Figure 4:**
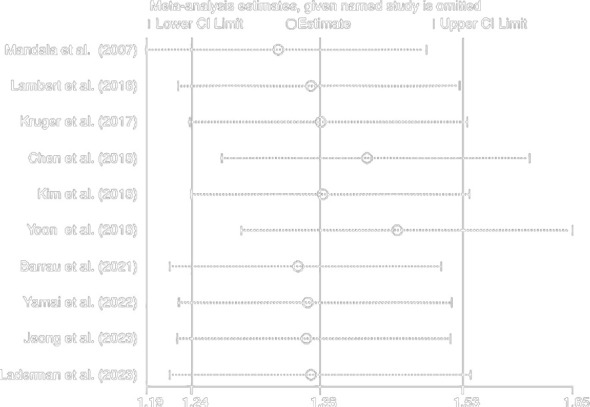
Sensitivity on the incidence of VTE and OS of patients.

**Figure 5:**
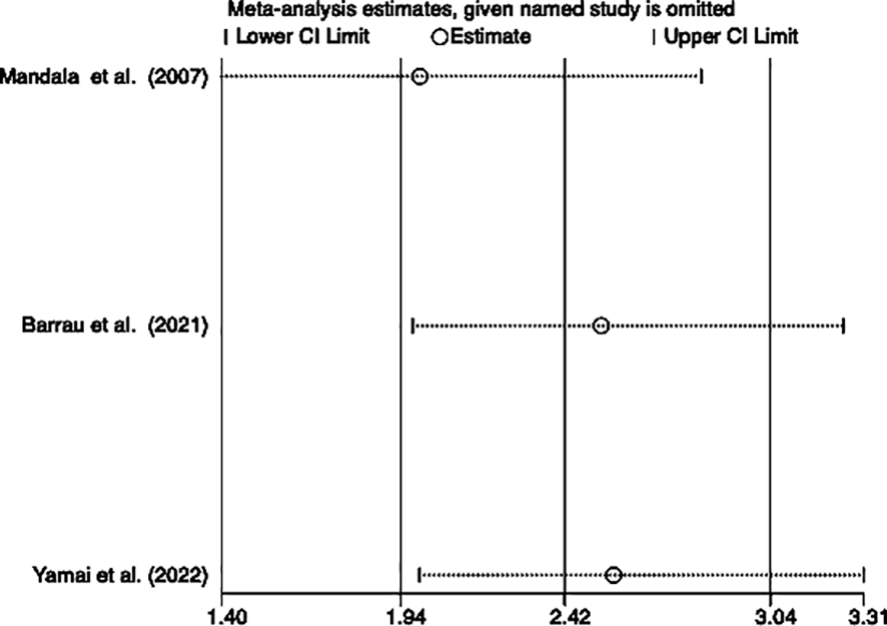
Sensitivity on the incidence of VTE and DFS of patients.

We performed a sensitivity analysis to observe whether a particular article influenced the overall analysis results. Consequently, the results of this meta-analysis turned out to be stable and reliable for the investigation into the relationship between OS and DFS (**Figures 4**, **5**).

### Publication bias

**Figure 6:**
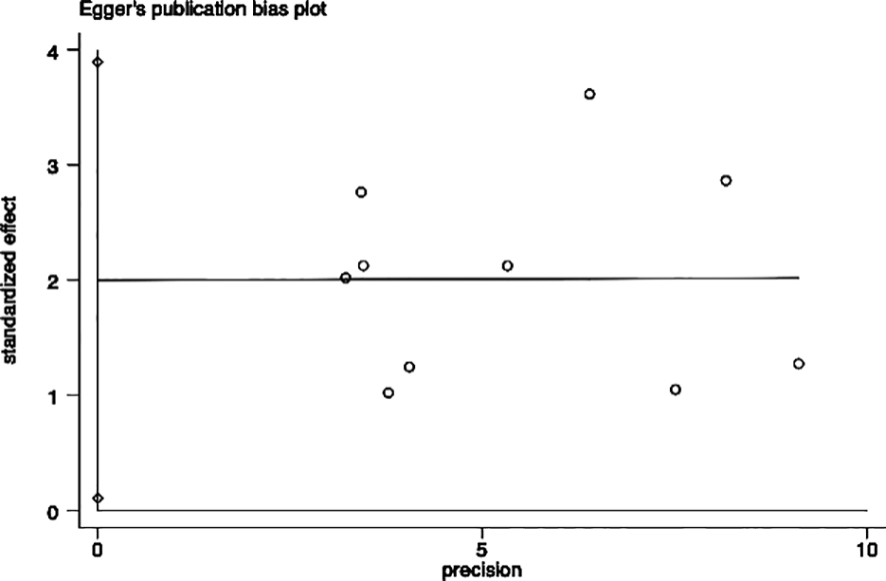
Egger test for evaluating the publication bias of this meta-analysis.

**Figure 7:**
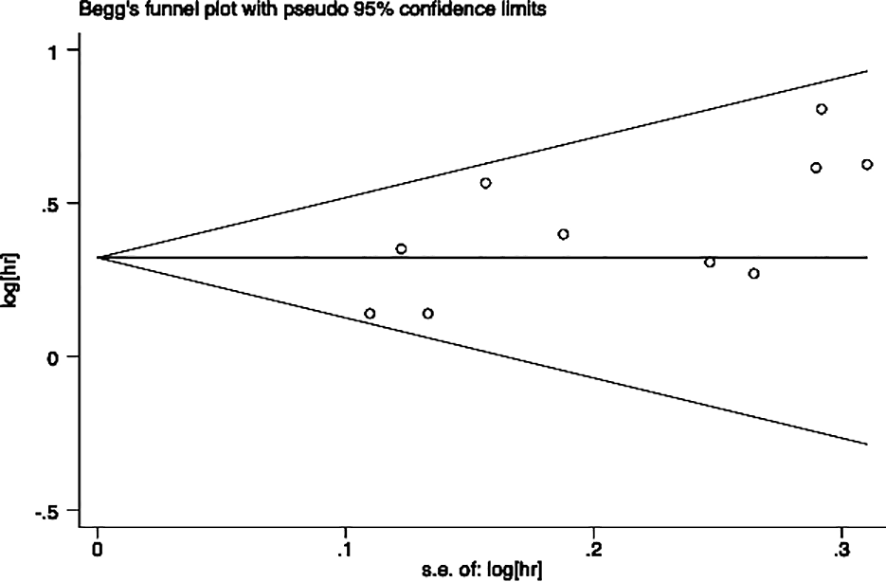
Begg test for evaluating the publication bias of this meta-analysis.

**Figure 8:**
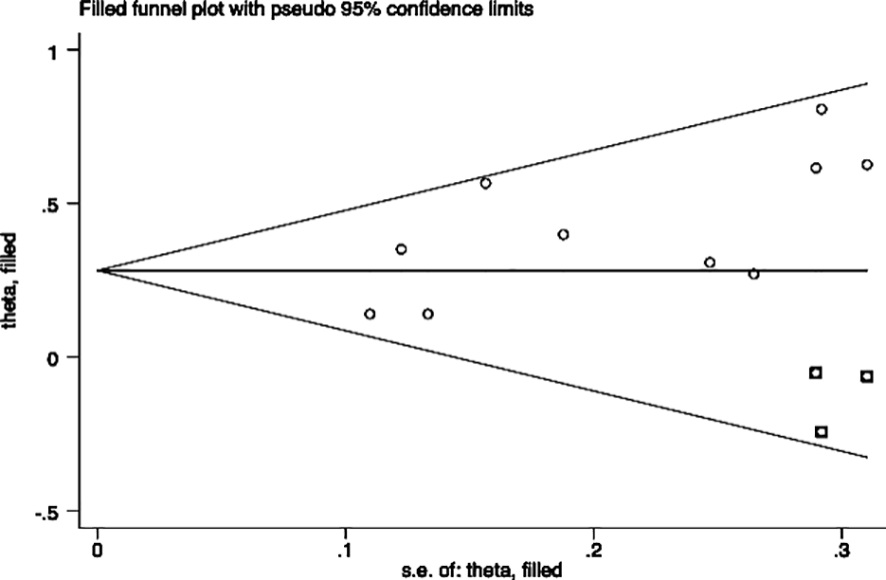
Funnel plot after Trim and Fill analysis.

Egger’s and Begg’s tests were performed to assess the presence of publication bias across included studies (**Figures 6**, **7**). Begg’s test yielded a p-value of 0.107, while the r’s test yielded a p-value of 0.041. These results suggested the possibility of publication bias. Therefore, the trim and fill method to was utilized assess the stability of the results. As a result, three articles were filled, and no changes were found in the results (P < 0.001) (**Figure 8**). In this regard, it can be concluded that publication bias did not significantly impact the final analysis results.

A comprehensive summary of the analyses and results can be found in **Table 2**.

## Discussion

In the present systematic review and meta-analysis, we identified a poorer prognosis in patients with advanced pancreatic cancer patients who experienced VTE than those without VTE. Several factors may contribute to this observation.

Biological experiments have demonstrated that pancreatic cancer cells can induce a prothrombotic state by expressing tissue factor or procoagulant extracellular vesicles to hinder physiological inhibitors of coagulation and directly activate platelets to prompt the formation of neutrophil extracellular traps (26–29). In contrast, it has been demonstrated that activation of the hemostatic system promotes the development and progression of pancreatic cancer by enhancing cancer cell proliferation, invasion, metastasis, immune evasion, and chemoresistance (30–32). Therefore, the activation of the hemostatic system may benefit the progression of pancreatic cancer. Moreover, it was reported that a high incidence of VTE was specifically correlated with early metastatic development and the malignant grade of tumors (33). These findings suggest a close relationship between VTE occurrence and the aggressiveness of pancreatic cancer.

Aside from the liver and lung, bone tissue is the most frequent site of hematogenous metastasis (34). The prognosis of patients can be significantly impacted by bone metastases, which can result in bone pain, pathological fractures, spinal cord compression, hypercalcemia, and other unpleasant symptoms (35). Some studies reported the occurrence of bone metastasis in pancreatic cancer and characterized by unfavorable outcomes. Skeletal metastases represent an underappreciated site of metastasis in patients with pancreatic cancer; however, the incidence of bone metastasis has increased in pancreatic cancer in recent years (36–39). Some patients with cancers were characterized by hypercoagulability and prone to thrombosis, which is the prerequisite for blood metastasis of tumor cells (40). Li et al. (41) studied that bone metastasis is more likely to occur when thrombosis occurs. Patients with bone metastasis were usually accompanied by impaired mobility, raising the incidence of VTE (42). Bone tumors could compress blood arteries, reducing venous blood flow, which increases the risk of vein thrombosis. Therefore, there may be a positive circuit between VTE and bone metastasis, both of which affect the prognosis of patients with pancreatic cancer. The particular mechanism needs to be researched in the future.

In subsequent subgroup analysis, patients were categorized into early and late VTE subgroups based on the timing of the onset of VTE. Interestingly, our findings revealed that only early VTE was associated with reduced patient survival, whereas late VTE did not exhibit the same impact. Generally, cancer cells generate procoagulant activators, such as tissue factor (TF), which trigger the coagulation cascade and contribute to an intrinsic and extrinsic hypercoagulable status, ultimately leading to the development of VTE (43). Studies have demonstrated that TF expression occurs early in the neoplastic transformation of pancreatic cancer. This expression is linked to vascular endothelial growth factor (VEGF) expression, enhanced vascular permeability, and increased micro-vessel density, contributing to heightened mitogenic activity (44). In this regard, we propose that early VTE during cancer diagnosis might indicate an enhanced angiogenic status of the tumor, suggesting biologically aggressive characteristics responsible for a short prognosis. Moreover, it has been found that a hypercoagulable state in the body is correlated with a poor response to chemotherapy (45). Additionally, early VTE detection poses challenges as it often occurs without noticeable symptoms, with two-thirds of patients with early VTE exhibiting asymptomatic VTE, leading to missed therapeutic opportunities (23). Hence, we recommend that VTE screening be conducted even in patients without VTE-related symptoms at the initiation of palliative chemotherapy. Furthermore, the increased mortality observed in patients with early VTE may also be attributed to additional morbidity resulting from VTE itself, interruptions or delays in chemotherapy due to VTE management, or the administration of anticoagulant therapy.

Our data analysis showed that VTE had a significant impact on the OS of patients, whether the data was directly extracted from papers or extracted from the survival curves in articles. This indicates that our results exhibited a reliable and acceptable level of heterogeneity. Furthermore, it was observed that VTE had a detrimental effect on the OS of patients in both Europe and America, as well as in Asia. Despite the common understanding that Asian patients have a lower incidence of VTE compared to Western patients due to genetic, environmental, and lifestyle factors (46, 47), our results highlight the importance of not neglecting the role of VTE in Asian patients, as the effects of VTE did not exhibit significant difference based on region or race.

All patients included in our research received palliative chemotherapy or chemo/radiotherapy. In both of these treatment subgroups, it was observed that VTE had similar effects on patient survival. Chemotherapy exposure is known to independently increase the risk of VTE in patients with pancreatic cancer, and cytotoxic drugs may damage endothelial cells, promote thrombus formation, and alter the expression of coagulation factors, thereby exacerbating the hypercoagulable state associated with tumors (48). Additionally, emerging evidence suggests that radiation could enhance a pro-coagulant response and induce primary hemostasis, potentially leading to thrombosis (33). This raises the question of whether anticoagulant treatment should be considered for patients with advanced pancreatic cancer undergoing palliative therapy. Some researchers propose a preventative strategy to mitigate thrombosis in patients with pancreatic cancer, recommending the use of low molecular weight heparin (LMWH) as a first-line option for primary VTE prevention over several weeks, and several clinical trials have shown that this approach is beneficial for patient survival (44, 49). In fact, some Japanese experts have even suggested that patients with pancreatic cancer should receive long-term anticoagulant therapy until the tumor is healed to minimize the risk of VTE recurrence (50). Therefore, further investigation is warranted to determine the optimal details of the future anticoagulant strategy for these patients.

This study has several strengths. Firstly, this is the first study conducted on the effect of VTE on the prognosis of advanced pancreatic cancer. Secondly, all of our included articles were studied over a more extended period, which adds strength of evidence for survival studies. Thirdly, we had all survival analyses, including the survival curves and HR (hazard ratio), and we also conducted subgroup analyses exploring the prognostic impact of VTE under different conditions.

Despite the valuable findings obtained in this research, several limitations should be acknowledged. First, the majority of our data stemmed from retrospective analyses, which inherently introduce the potential for selection bias. Additionally, due to the lack of individual survival data associated with types of VTE and drugs in the original articles, we were unable to specifically classify the types of VTE or the specific palliative treatment drugs. Third, the absence of unified criteria or guidelines to define the timing of early VTE occurrence may have introduced calculation errors, resulting in deviations in the outcomes.

## Conclusion

Our analysis collectively demonstrated that VTE predicted a poor prognosis in advanced pancreatic cancer when patients had it. Notably, patients who had early VTE experienced a considerably worse prognosis, but those with late VTE did not in the subgroup analysis. These findings hold partial value for informing further clinical work and reminding clinicians about the crucial role of early VTE detection prior to treatment initiation. it is essential to emphasize the necessity for future multicenter, large-scale, and prospective studies to elucidate the true significance and implications of these findings.

## Data availability statement

The original contributions presented in the study are included in the article/**Supplementary Material**. Further inquiries can be directed to the corresponding author.

## Author contributions

KS: Writing – original draft, Writing – review & editing. RD: Writing – review & editing. YW: Writing – review & editing.
