# Supplementary material for: Prognostic value of venous thromboembolism in patients with advanced pancreatic cancer: a systematic review and meta-analysis
Source: Front Oncol. 2024 Feb 8;14:1331706. doi: 10.3389/fonc.2024.1331706 (PMC10882063; doi:10.3389/fonc.2024.1331706)
Supplement: Supplementary file 1 [file DataSheet_1.pdf]

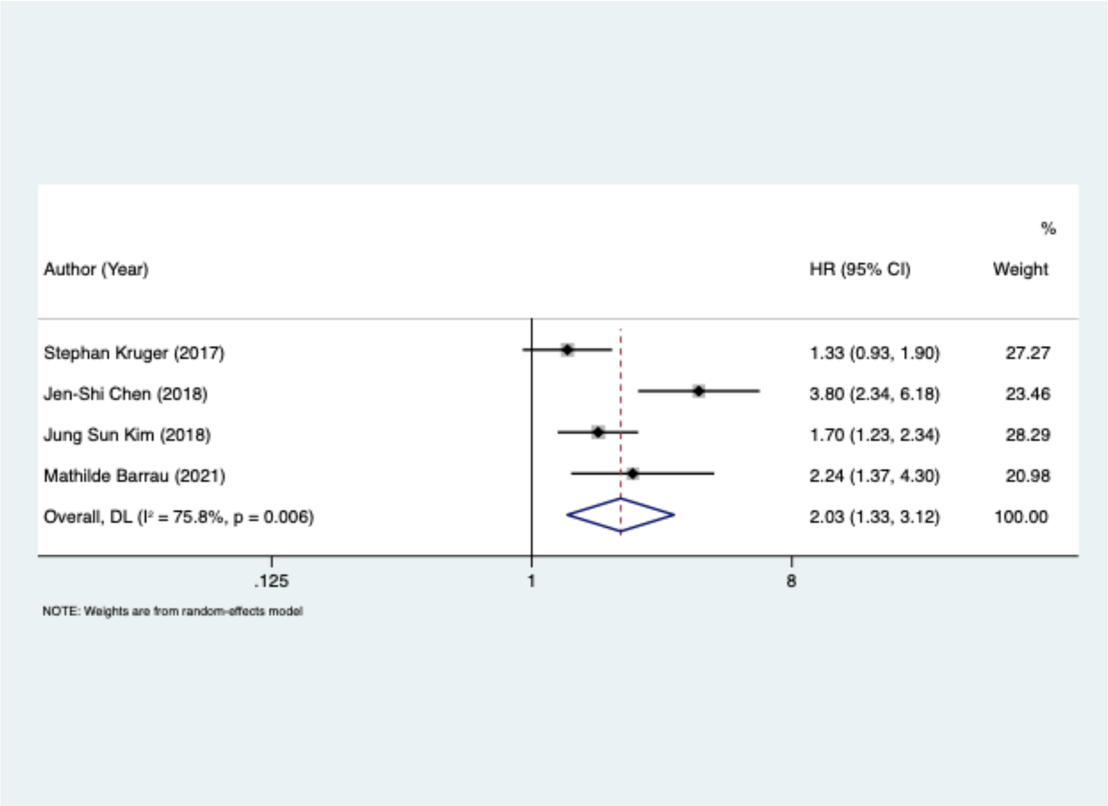

Figure S1. Forest plots of 4 studies examining the association between early VTE and the OS of patients with advanced pancreatic cancer.

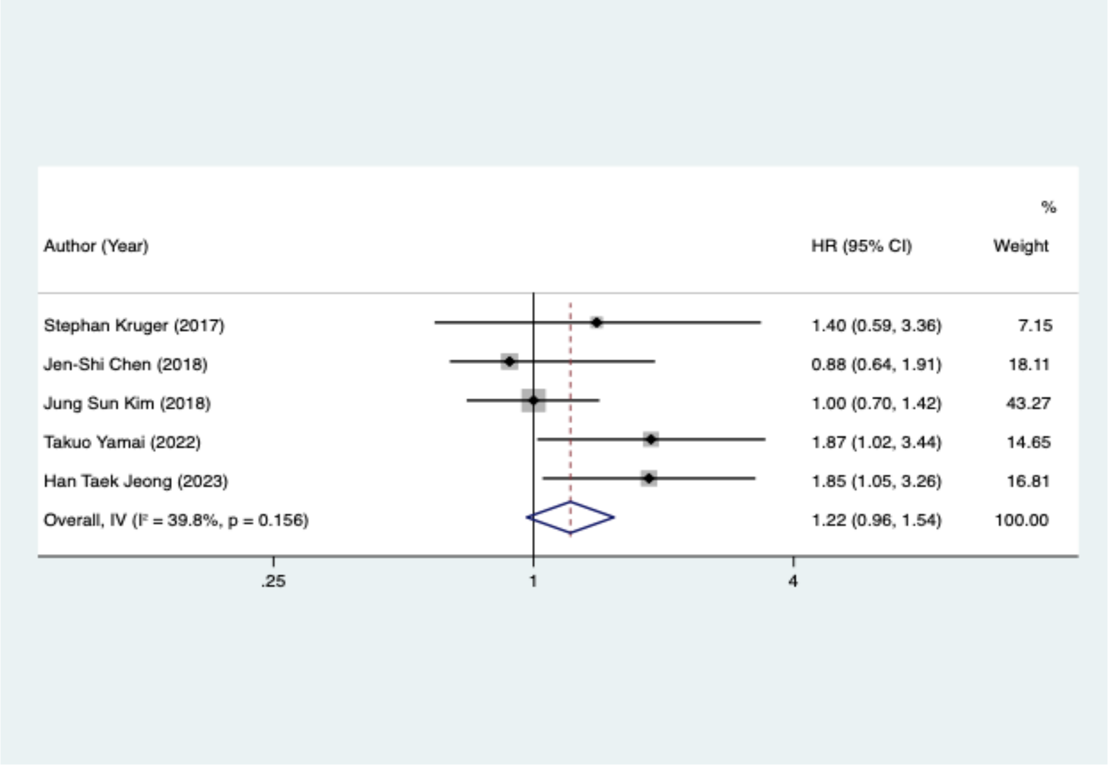

Figure S2. Forest plots of 5 studies examining the association between late VTE and the OS of patients with advanced pancreatic cancer.

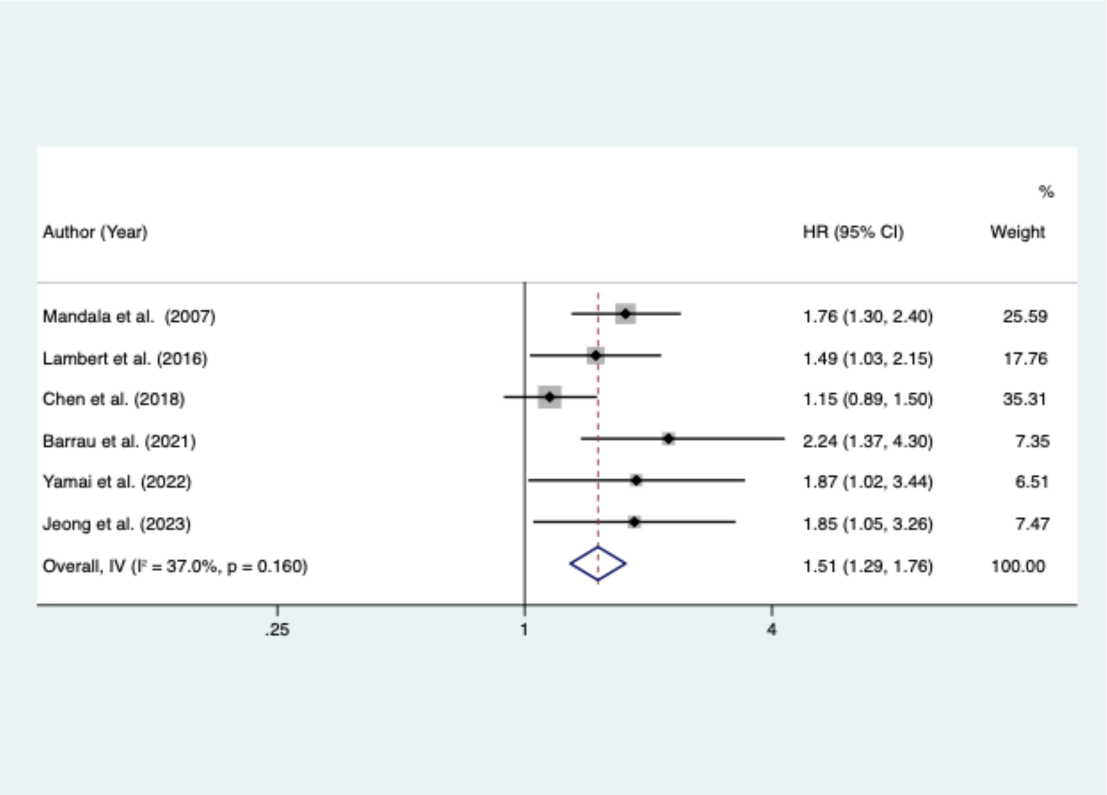

Figure S3. Forest plots of 6 studies examining the association between VTE and the OS of patients with advanced pancreatic cancer through HR (95% CI).

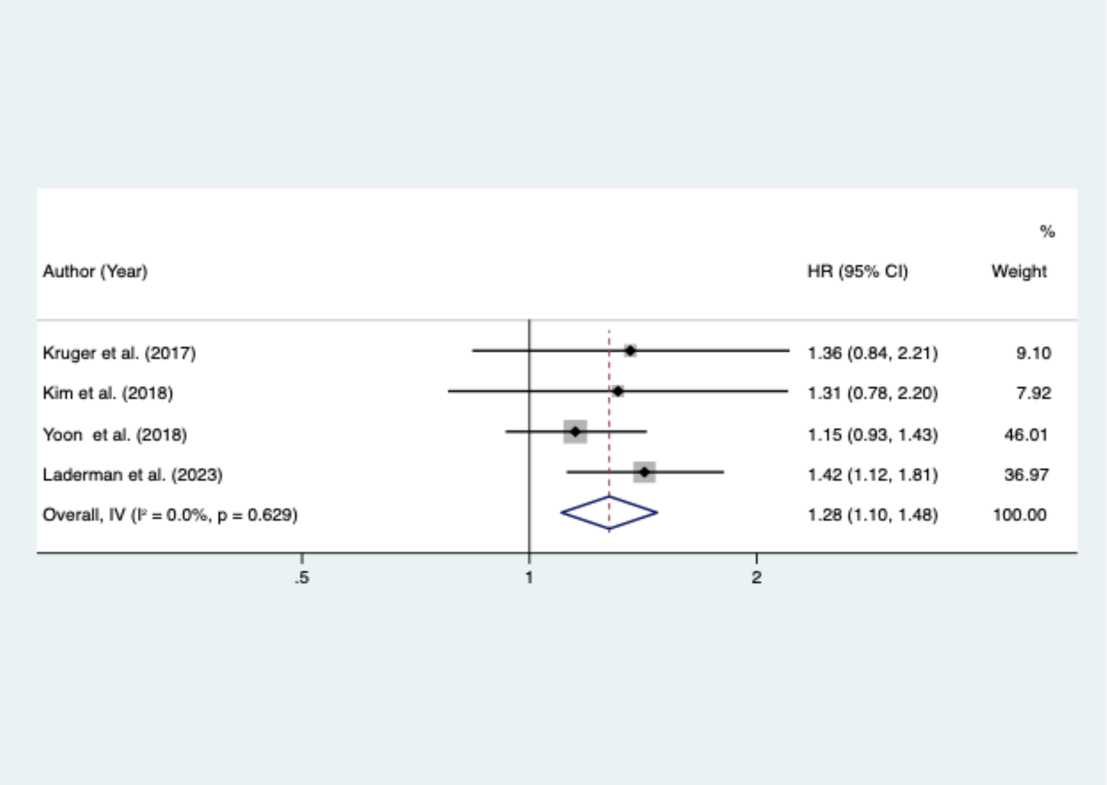

Figure S4. Forest plots of 4 studies examining the association between VTE and the OS of patients with advanced pancreatic cancer through survival curves.

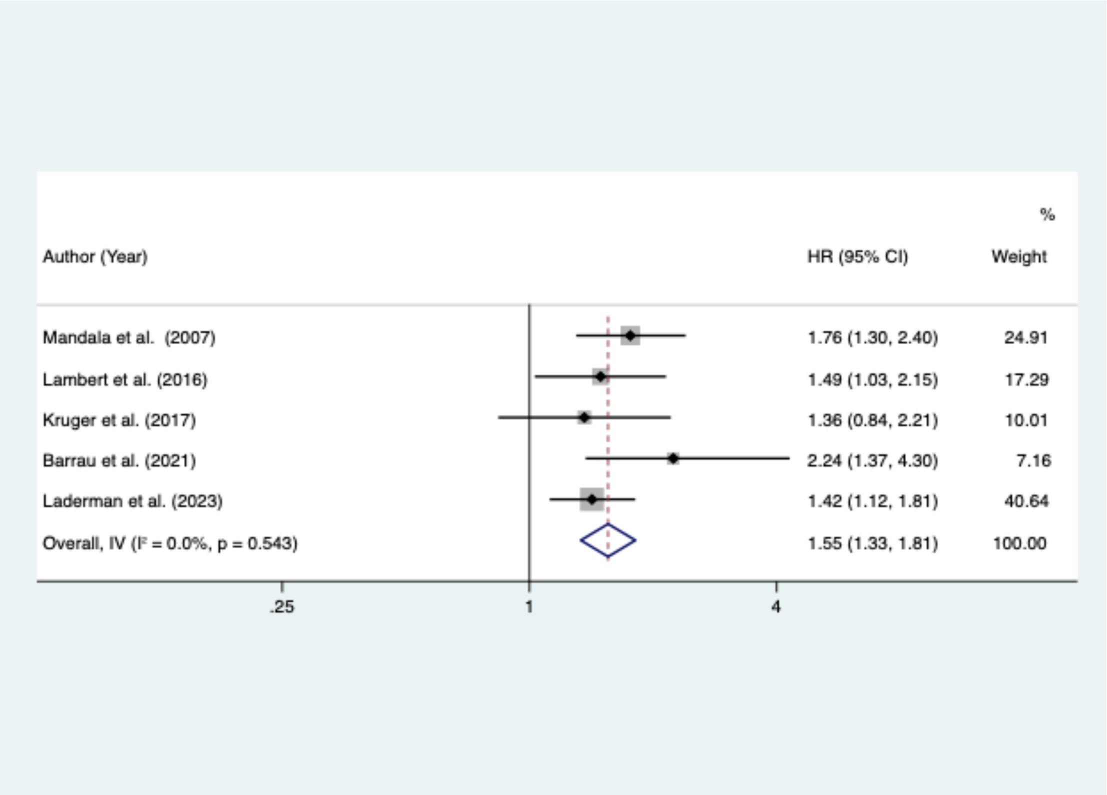

Figure S5. Forest plots of 5 studies examining the association between VTE and the OS of patients with advanced pancreatic cancer in Europe and America.

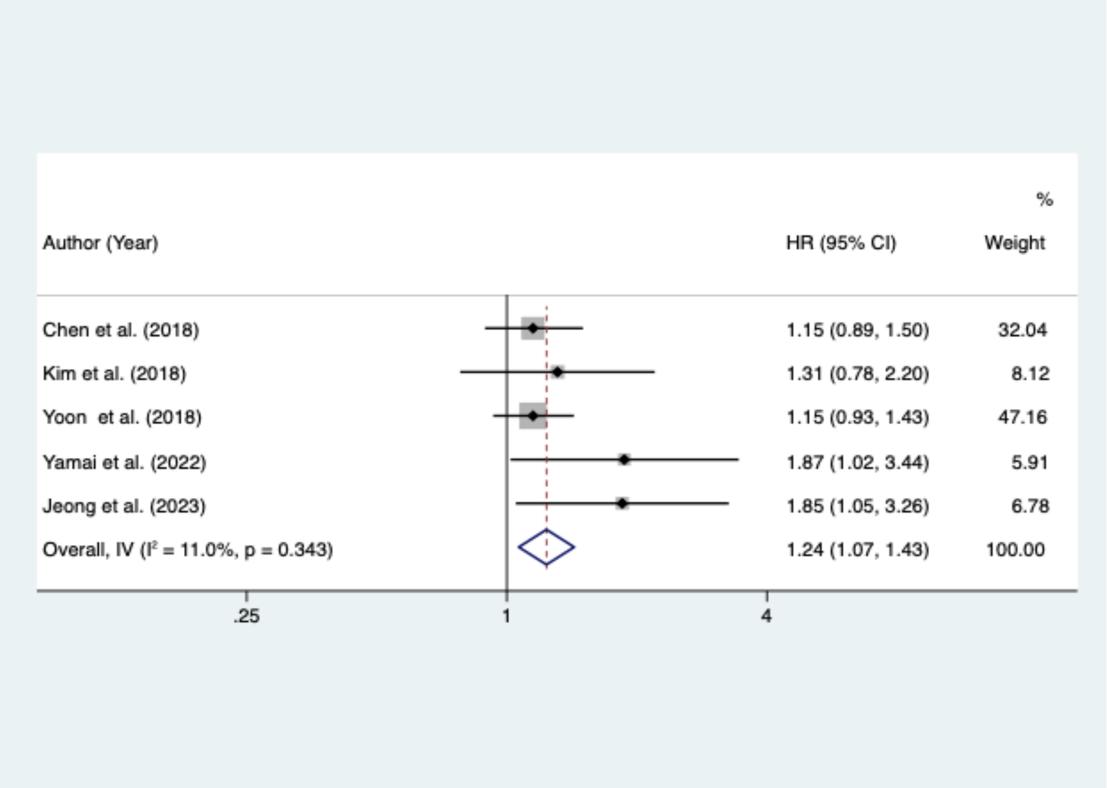

Figure S6. Forest plots of 5 studies examining the association between VTE and the OS of patients with advanced pancreatic cancer in Asia.

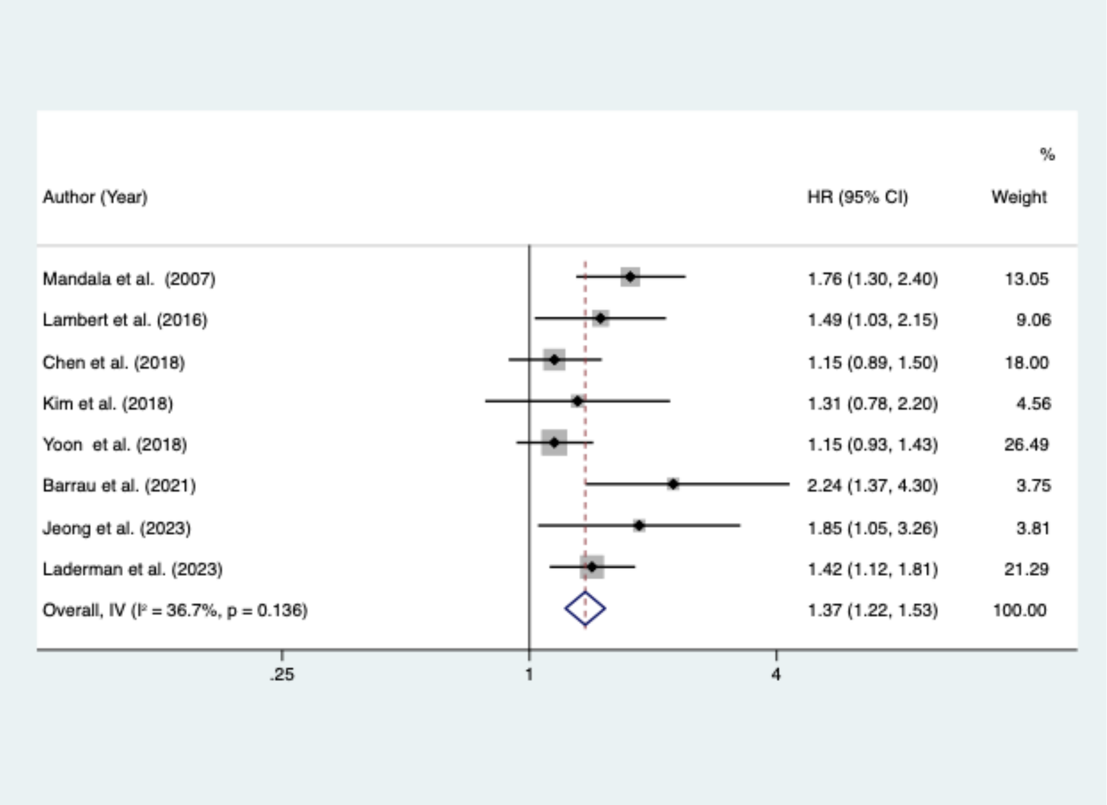

Figure S7. Forest plots of 8 studies examining the association between VTE and the OS of patients with advanced pancreatic cancer in chemotherapy group.

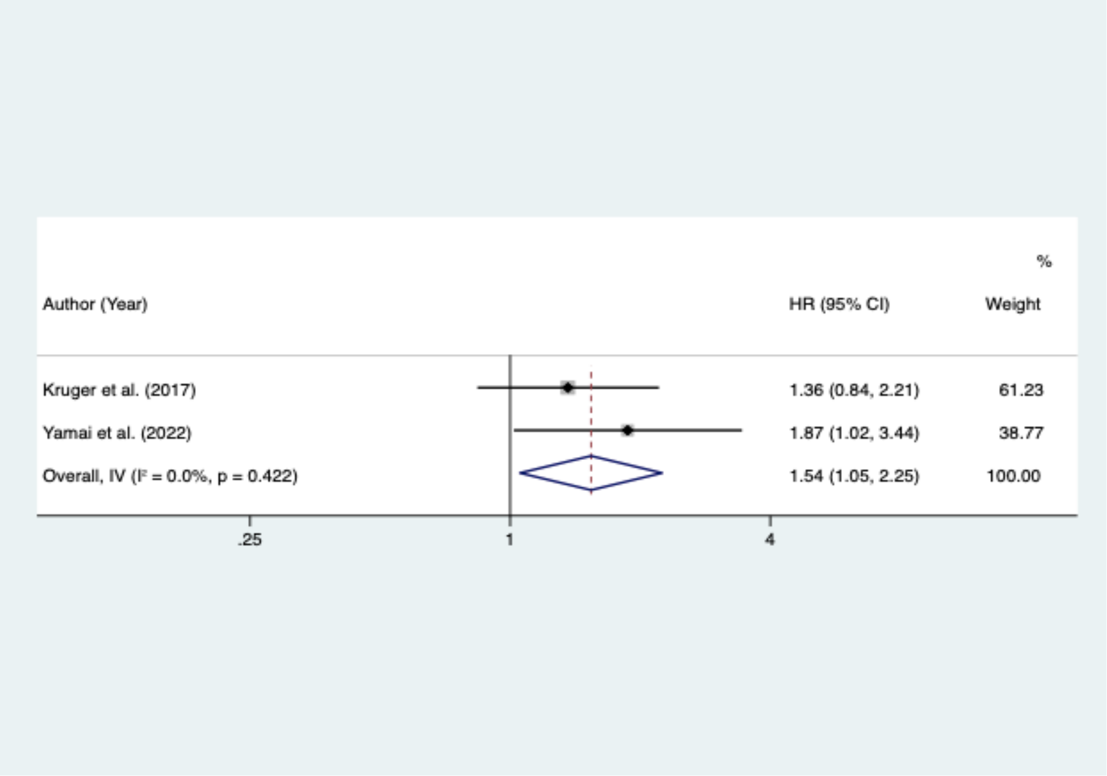

Figure S8. Forest plots of 2 studies examining the association between VTE and the OS of patients with advanced pancreatic cancer in chemoradiotherapy group.
