# Supplementary material for: Prognostic value of venous thromboembolism in patients with advanced pancreatic cancer: a systematic review and meta-analysis
Source: Front Oncol. 2024 Feb 8;14:1331706. doi: 10.3389/fonc.2024.1331706 (PMC10882063; doi:10.3389/fonc.2024.1331706)
Supplement: Supplementary file 2 [file Table_1.docx]

**Pubmed**

| **Search Number** | **Search Strategy** |
| --- | --- |
| 1 | "Pancreatic Neoplasms"[Mesh] |
| 2 | (((((((((((((((((((Pancreatic Neoplasms[Title/Abstract]) OR (Neoplasm, Pancreatic[Title/Abstract])) OR (Pancreatic Neoplasm[Title/Abstract])) OR (Pancreas Neoplasms[Title/Abstract])) OR (Neoplasm, Pancreas[Title/Abstract])) OR (Neoplasms, Pancreas[Title/Abstract])) OR (Pancreas Neoplasm[Title/Abstract])) OR (Neoplasms, Pancreatic[Title/Abstract])) OR (Cancer of Pancreas[Title/Abstract])) OR (Pancreas Cancers[Title/Abstract])) OR (Pancreas Cancer[Title/Abstract])) OR (Cancer, Pancreas[Title/Abstract])) OR (Cancers, Pancreas[Title/Abstract])) OR (Pancreatic Cancer[Title/Abstract])) OR (Cancer, Pancreatic[Title/Abstract])) OR (Cancers, Pancreatic[Title/Abstract])) OR (Pancreatic Cancers[Title/Abstract])) OR (Cancer of the Pancreas[Title/Abstract])) OR (Pancreatic adenocarcinoma[Title/Abstract])) OR (Pancreatic ductal adenocarcinoma[Title/Abstract]) |
| 3 | ("Pancreatic Neoplasms"[Mesh]) OR ((((((((((((((((((((Pancreatic Neoplasms[Title/Abstract]) OR (Neoplasm, Pancreatic[Title/Abstract])) OR (Pancreatic Neoplasm[Title/Abstract])) OR (Pancreas Neoplasms[Title/Abstract])) OR (Neoplasm, Pancreas[Title/Abstract])) OR (Neoplasms, Pancreas[Title/Abstract])) OR (Pancreas Neoplasm[Title/Abstract])) OR (Neoplasms, Pancreatic[Title/Abstract])) OR (Cancer of Pancreas[Title/Abstract])) OR (Pancreas Cancers[Title/Abstract])) OR (Pancreas Cancer[Title/Abstract])) OR (Cancer, Pancreas[Title/Abstract])) OR (Cancers, Pancreas[Title/Abstract])) OR (Pancreatic Cancer[Title/Abstract])) OR (Cancer, Pancreatic[Title/Abstract])) OR (Cancers, Pancreatic[Title/Abstract])) OR (Pancreatic Cancers[Title/Abstract])) OR (Cancer of the Pancreas[Title/Abstract])) OR (Pancreatic adenocarcinoma[Title/Abstract])) OR (Pancreatic ductal adenocarcinoma[Title/Abstract])) |
| 4 | "Venous Thrombosis"[Mesh] |
| 5 | (((((((((((((((((((((((((Venous Thrombosis[Title/Abstract]) OR (Phlebothrombosis[Title/Abstract])) OR (Phlebothromboses[Title/Abstract])) OR (Thrombosis, Venous[Title/Abstract])) OR (Thromboses, Venous[Title/Abstract])) OR (Venous Thromboses[Title/Abstract])) OR (Deep Vein Thrombosis[Title/Abstract])) OR (Deep Vein Thromboses[Title/Abstract])) OR (Thromboses, Deep Vein[Title/Abstract])) OR (Vein Thromboses, Deep[Title/Abstract])) OR (Vein Thrombosis, Deep[Title/Abstract])) OR (Deep-Venous Thrombosis[Title/Abstract])) OR (Deep-Venous Thromboses[Title/Abstract])) OR (Thromboses, Deep-Venous[Title/Abstract])) OR (Thrombosis, Deep-Venous[Title/Abstract])) OR (Deep-Vein Thrombosis[Title/Abstract])) OR (Deep-Vein Thromboses[Title/Abstract])) OR (Thromboses, Deep-Vein[Title/Abstract])) OR (Thrombosis, Deep-Vein[Title/Abstract])) OR (Thrombosis, Deep Vein[Title/Abstract])) OR (Deep Venous Thrombosis[Title/Abstract])) OR (Deep Venous Thromboses[Title/Abstract])) OR (Thromboses, Deep Venous[Title/Abstract])) OR (Thrombosis, Deep Venous[Title/Abstract])) OR (Venous Thromboses, Deep[Title/Abstract])) OR (Venous Thrombosis, Deep[Title/Abstract]) |
| 6 | ("Venous Thrombosis"[Mesh]) OR ((((((((((((((((((((((((((Venous Thrombosis[Title/Abstract]) OR (Phlebothrombosis[Title/Abstract])) OR (Phlebothromboses[Title/Abstract])) OR (Thrombosis, Venous[Title/Abstract])) OR (Thromboses, Venous[Title/Abstract])) OR (Venous Thromboses[Title/Abstract])) OR (Deep Vein Thrombosis[Title/Abstract])) OR (Deep Vein Thromboses[Title/Abstract])) OR (Thromboses, Deep Vein[Title/Abstract])) OR (Vein Thromboses, Deep[Title/Abstract])) OR (Vein Thrombosis, Deep[Title/Abstract])) OR (Deep-Venous Thrombosis[Title/Abstract])) OR (Deep-Venous Thromboses[Title/Abstract])) OR (Thromboses, Deep-Venous[Title/Abstract])) OR (Thrombosis, Deep-Venous[Title/Abstract])) OR (Deep-Vein Thrombosis[Title/Abstract])) OR (Deep-Vein Thromboses[Title/Abstract])) OR (Thromboses, Deep-Vein[Title/Abstract])) OR (Thrombosis, Deep-Vein[Title/Abstract])) OR (Thrombosis, Deep Vein[Title/Abstract])) OR (Deep Venous Thrombosis[Title/Abstract])) OR (Deep Venous Thromboses[Title/Abstract])) OR (Thromboses, Deep Venous[Title/Abstract])) OR (Thrombosis, Deep Venous[Title/Abstract])) OR (Venous Thromboses, Deep[Title/Abstract])) OR (Venous Thrombosis, Deep[Title/Abstract])) |
| 7 | "Venous Thromboembolism"[Mesh] |
| 8 | (((Venous Thromboembolism[Title/Abstract]) OR (Thromboembolism, Venous[Title/Abstract])) OR (Thromboembolic[Title/Abstract])) OR (Thrombogenesis[Title/Abstract]) |
| 9 | ("Venous Thromboembolism"[Mesh]) OR ((((Venous Thromboembolism[Title/Abstract]) OR (Thromboembolism, Venous[Title/Abstract])) OR (Thromboembolic[Title/Abstract])) OR (Thrombogenesis[Title/Abstract])) |
| 10 | (("Venous Thrombosis"[Mesh]) OR ((((((((((((((((((((((((((Venous Thrombosis[Title/Abstract]) OR (Phlebothrombosis[Title/Abstract])) OR (Phlebothromboses[Title/Abstract])) OR (Thrombosis, Venous[Title/Abstract])) OR (Thromboses, Venous[Title/Abstract])) OR (Venous Thromboses[Title/Abstract])) OR (Deep Vein Thrombosis[Title/Abstract])) OR (Deep Vein Thromboses[Title/Abstract])) OR (Thromboses, Deep Vein[Title/Abstract])) OR (Vein Thromboses, Deep[Title/Abstract])) OR (Vein Thrombosis, Deep[Title/Abstract])) OR (Deep-Venous Thrombosis[Title/Abstract])) OR (Deep-Venous Thromboses[Title/Abstract])) OR (Thromboses, Deep-Venous[Title/Abstract])) OR (Thrombosis, Deep-Venous[Title/Abstract])) OR (Deep-Vein Thrombosis[Title/Abstract])) OR (Deep-Vein Thromboses[Title/Abstract])) OR (Thromboses, Deep-Vein[Title/Abstract])) OR (Thrombosis, Deep-Vein[Title/Abstract])) OR (Thrombosis, Deep Vein[Title/Abstract])) OR (Deep Venous Thrombosis[Title/Abstract])) OR (Deep Venous Thromboses[Title/Abstract])) OR (Thromboses, Deep Venous[Title/Abstract])) OR (Thrombosis, Deep Venous[Title/Abstract])) OR (Venous Thromboses, Deep[Title/Abstract])) OR (Venous Thrombosis, Deep[Title/Abstract]))) OR (("Venous Thromboembolism"[Mesh]) OR ((((Venous Thromboembolism[Title/Abstract]) OR (Thromboembolism, Venous[Title/Abstract])) OR (Thromboembolic[Title/Abstract])) OR (Thrombogenesis[Title/Abstract]))) |
| 11 | (("Pancreatic Neoplasms"[Mesh]) OR ((((((((((((((((((((Pancreatic Neoplasms[Title/Abstract]) OR (Neoplasm, Pancreatic[Title/Abstract])) OR (Pancreatic Neoplasm[Title/Abstract])) OR (Pancreas Neoplasms[Title/Abstract])) OR (Neoplasm, Pancreas[Title/Abstract])) OR (Neoplasms, Pancreas[Title/Abstract])) OR (Pancreas Neoplasm[Title/Abstract])) OR (Neoplasms, Pancreatic[Title/Abstract])) OR (Cancer of Pancreas[Title/Abstract])) OR (Pancreas Cancers[Title/Abstract])) OR (Pancreas Cancer[Title/Abstract])) OR (Cancer, Pancreas[Title/Abstract])) OR (Cancers, Pancreas[Title/Abstract])) OR (Pancreatic Cancer[Title/Abstract])) OR (Cancer, Pancreatic[Title/Abstract])) OR (Cancers, Pancreatic[Title/Abstract])) OR (Pancreatic Cancers[Title/Abstract])) OR (Cancer of the Pancreas[Title/Abstract])) OR (Pancreatic adenocarcinoma[Title/Abstract])) OR (Pancreatic ductal adenocarcinoma[Title/Abstract]))) AND ((("Venous Thrombosis"[Mesh]) OR ((((((((((((((((((((((((((Venous Thrombosis[Title/Abstract]) OR (Phlebothrombosis[Title/Abstract])) OR (Phlebothromboses[Title/Abstract])) OR (Thrombosis, Venous[Title/Abstract])) OR (Thromboses, Venous[Title/Abstract])) OR (Venous Thromboses[Title/Abstract])) OR (Deep Vein Thrombosis[Title/Abstract])) OR (Deep Vein Thromboses[Title/Abstract])) OR (Thromboses, Deep Vein[Title/Abstract])) OR (Vein Thromboses, Deep[Title/Abstract])) OR (Vein Thrombosis, Deep[Title/Abstract])) OR (Deep-Venous Thrombosis[Title/Abstract])) OR (Deep-Venous Thromboses[Title/Abstract])) OR (Thromboses, Deep-Venous[Title/Abstract])) OR (Thrombosis, Deep- |
|  | Venous[Title/Abstract])) OR (Deep-Vein Thrombosis[Title/Abstract])) OR (Deep-Vein Thromboses[Title/Abstract])) OR (Thromboses, Deep-Vein[Title/Abstract])) OR (Thrombosis, Deep-Vein[Title/Abstract])) OR (Thrombosis, Deep Vein[Title/Abstract])) OR (Deep Venous Thrombosis[Title/Abstract])) OR (Deep Venous Thromboses[Title/Abstract])) OR (Thromboses, Deep Venous[Title/Abstract])) OR (Thrombosis, Deep Venous[Title/Abstract])) OR (Venous Thromboses, Deep[Title/Abstract])) OR (Venous Thrombosis, Deep[Title/Abstract]))) OR (("Venous Thromboembolism"[Mesh]) OR ((((Venous Thromboembolism[Title/Abstract]) OR (Thromboembolism, Venous[Title/Abstract])) OR (Thromboembolic[Title/Abstract])) OR (Thrombogenesis[Title/Abstract])))) |

**Embase**

| **Search Number** | **Search Strategy** |
| --- | --- |
| #1 | 'pancreas tumor'/exp |
| #2 | 'pancreatic neoplasms':ab,ti OR 'neoplasm, pancreatic':ab,ti OR 'pancreatic neoplasm':ab,ti OR 'pancreas neoplasms':ab,ti OR 'neoplasm, pancreas':ab,ti OR 'neoplasms, pancreas':ab,ti OR 'pancreas neoplasm':ab,ti OR 'neoplasms, pancreatic':ab,ti OR 'cancer of pancreas':ab,ti OR 'pancreas cancers':ab,ti OR 'pancreas cancer':ab,ti OR 'cancer, pancreas':ab,ti OR 'cancers, pancreas':ab,ti OR 'pancreatic cancer':ab,ti OR 'cancer, pancreatic':ab,ti OR 'cancers, pancreatic':ab,ti OR 'pancreatic cancers':ab,ti OR 'cancer of the pancreas':ab,ti OR 'pancreatic adenocarcinoma':ab,ti OR 'pancreatic ductal adenocarcinoma':ab,ti |
| #3 | #1 OR #2 |
| #4 | 'vein thrombosis'/exp |
| #5 | 'venous thrombosis':ab,ti OR phlebothrombosis:ab,ti OR phlebothromboses:ab,ti OR 'thrombosis, venous':ab,ti OR 'thromboses, venous':ab,ti OR 'venous thromboses':ab,ti OR 'deep vein thrombosis':ab,ti OR 'deep vein thromboses':ab,ti OR 'thromboses, deep vein':ab,ti OR 'vein thromboses, deep':ab,ti OR 'vein thrombosis, deep':ab,ti OR 'deep-venous thrombosis':ab,ti OR 'deep-venous thromboses':ab,ti OR 'thromboses, deep-venous':ab,ti OR 'thrombosis, deep-venous':ab,ti OR 'deep-vein thrombosis':ab,ti OR 'deep-vein thromboses':ab,ti OR 'thromboses, deep-vein':ab,ti OR 'thrombosis, deep-vein':ab,ti OR 'thrombosis, deep vein':ab,ti OR 'deep venous thrombosis':ab,ti OR 'deep venous thromboses':ab,ti OR 'thromboses, deep venous':ab,ti OR 'thrombosis, deep venous':ab,ti OR 'venous thromboses, deep':ab,ti OR 'venous thrombosis, deep':ab,ti |
| #6 | #4 OR #5 |
| #7 | 'venous thromboembolism'/exp |
| #8 | 'venous thromboembolism':ab,ti OR 'thromboembolism, venous':ab,ti OR thromboembolic:ab,ti OR thrombogenesis:ab,ti |
| #9 | #7 OR #8 |
| #10 | #6 OR #9 |
| #11 | #3 AND #10 |

**Cochrane**

| **Search Number** | **Search Strategy** |
| --- | --- |
| #1 | MeSH descriptor: [Pancreatic Neoplasms] explode all trees |
| #2 | (Pancreatic Neoplasms):ti,ab,kw OR (Neoplasm, Pancreatic):ti,ab,kw OR (Pancreatic Neoplasm):ti,ab,kw OR (Pancreas Neoplasms):ti,ab,kw OR (Neoplasm, Pancreas):ti,ab,kw |
| #3 | (Neoplasms, Pancreas):ti,ab,kw OR (Pancreas Neoplasm):ti,ab,kw OR (Neoplasms, Pancreatic):ti,ab,kw OR (Cancer of Pancreas):ti,ab,kw OR (Pancreas Cancers):ti,ab,kw |
| #4 | (Pancreas Cancer):ti,ab,kw OR (Cancer, Pancreas):ti,ab,kw OR (Cancers, Pancreas):ti,ab,kw OR (Pancreatic Cancer):ti,ab,kw OR (Cancer, Pancreatic):ti,ab,kw |
| #5 | (Cancers, Pancreatic):ti,ab,kw OR (Pancreatic Cancers):ti,ab,kw OR (Cancer of the Pancreas):ti,ab,kw OR (Pancreatic adenocarcinoma):ti,ab,kw OR (Pancreatic ductal adenocarcinoma):ti,ab,kw |
| #6 | #2 OR #3 OR #4 OR #5 |
| #7 | #1 OR #6 |
| #8 | MeSH descriptor: [Venous Thrombosis] explode all trees |
| #9 | (Venous Thrombosis):ti,ab,kw OR (Phlebothrombosis):ti,ab,kw OR (Phlebothromboses):ti,ab,kw OR (Thrombosis, Venous):ti,ab,kw OR (Thromboses, Venous):ti,ab,kw |
| #10 | (Venous Thromboses):ti,ab,kw OR (Deep Vein Thrombosis):ti,ab,kw OR (Deep Vein Thromboses):ti,ab,kw OR (Thromboses, Deep Vein):ti,ab,kw OR (Vein Thromboses, Deep):ti,ab,kw |
| #11 | (Vein Thrombosis, Deep):ti,ab,kw OR (Deep-Venous Thrombosis):ti,ab,kw OR (Deep-Venous Thromboses):ti,ab,kw OR (Thromboses, Deep-Venous):ti,ab,kw OR (Thrombosis, Deep-Venous):ti,ab,kw |
| #12 | (Deep-Vein Thrombosis):ti,ab,kw OR (Deep-Vein Thromboses):ti,ab,kw OR (Thromboses, Deep-Vein):ti,ab,kw OR (Thrombosis, Deep-Vein):ti,ab,kw OR (Thrombosis, Deep Vein):ti,ab,kw |
| #13 | (Deep Venous Thrombosis):ti,ab,kw OR (Deep Venous Thromboses):ti,ab,kw OR (Thromboses, Deep Venous):ti,ab,kw OR (Thrombosis, Deep Venous):ti,ab,kw OR (Venous Thromboses, Deep):ti,ab,kw |
| #14 | (Venous Thrombosis, Deep):ti,ab,kw |
| #15 | #9 OR #10 OR #11 OR #12 OR #13 OR #14 |
| #16 | #8 OR #15 |
| #17 | MeSH descriptor: [Venous Thromboembolism] explode all trees |
| #18 | (Venous Thromboembolism):ti,ab,kw OR (Thromboembolism, Venous):ti,ab,kw OR (Thromboembolic):ti,ab,kw OR (Thrombogenesis):ti,ab,kw |
| #19 | #17 OR #18 |
| #20 | #16 OR #19 |
| #21 | #7 AND #20 |

**Web Of Science**

| **Search Number** | **Search Strategy** |
| --- | --- |
| #1 | Topic: (Pancreatic Neoplasms) OR Topic: (Neoplasm, Pancreatic) OR Topic: (Pancreatic Neoplasm) OR Topic: (Pancreas Neoplasms) OR Topic: (Neoplasm, Pancreas) OR Topic: (Neoplasms, Pancreas) OR Topic: (Pancreas Neoplasm) OR Topic: (Neoplasms, Pancreatic) OR Topic: (Cancer of Pancreas) OR Topic: (Pancreas Cancers) OR Topic: (Pancreas Cancer) OR Topic: (Cancer, Pancreas) OR Topic: (Cancers, Pancreas) OR Topic: (Pancreatic Cancer) OR Topic: (Cancer, Pancreatic) OR Topic: (Cancers, Pancreatic) OR Topic: (Pancreatic Cancers) OR Topic: (Cancer of the Pancreas) OR Topic: (Pancreatic adenocarcinoma) OR Topic: (Pancreatic ductal adenocarcinoma) |
| #2 | Topic: (Venous Thrombosis) OR Topic: (Phlebothrombosis) OR Topic: (Phlebothromboses) OR Topic: (Thrombosis, Venous) OR Topic: (Thromboses, Venous) OR Topic: (Venous Thromboses) OR Topic: (Deep Vein Thrombosis) OR Topic: (Deep Vein Thromboses) OR Topic: (Thromboses, Deep Vein) OR Topic: (Vein Thromboses, Deep) OR Topic: (Vein Thrombosis, Deep) OR Topic: (Deep-Venous Thrombosis) OR Topic: (Deep-Venous Thromboses) OR Topic: (Thromboses, Deep-Venous) OR Topic: (Thrombosis, Deep-Venous) OR Topic: (Deep-Vein Thrombosis) OR Topic: (Deep-Vein Thromboses) OR Topic: (Thromboses, Deep-Vein) OR Topic: (Thrombosis, Deep-Vein) OR Topic: (Thrombosis, Deep Vein) OR Topic: (Deep Venous Thrombosis) OR Topic: (Deep Venous Thromboses) OR Topic: (Thromboses, Deep Venous) OR Topic: (Thrombosis, Deep Venous) OR Topic: (Venous Thromboses, Deep) |
| #3 | Topic: (Venous Thrombosis, Deep) OR Topic: (Venous Thromboembolism) OR Topic: (Thromboembolism, Venous) OR Topic: (Thromboembolic) OR Topic: (Thrombogenesis) |
| #4 | #2 OR #3 |
| #5 | #4 AND #1 |
